# Certain Dietary Nutrients Reduce the Risk of Eye Affliction/Retinopathy in Individuals with Diabetes: National Health and Nutrition Examination Survey, 2003–2018

**DOI:** 10.3390/ijerph191912173

**Published:** 2022-09-26

**Authors:** Guoheng Zhang, Xiaojia Sun, Tianhao Yuan, Changmei Guo, Ziyi Zhou, Ling Wang, Guorui Dou

**Affiliations:** 1 Department of Ophthalmology, Eye Institute of Chinese PLA, Xijing Hospital, Fourth Military Medical University, Xi’an 710032, China; 2 Department of Ophthalmology, 942 Hospital of the Joint Logistics Support Force of the Chinese People’s Liberation Army, Yinchuan 750000, China; 3 Department of Health Statistics, Faculty of Preventive Medicine, Fourth Military Medical University, Xi’an 710032, China

**Keywords:** diabetic eye disease, dietary nutrients, diabetic retinopathy, NHANES

## Abstract

As the global trend of diabetes intensifies, the burden of vision-threatening retinopathy, particularly diabetic retinopathy (DR), is increasing. There is an urgent need to seek strategies for early prevention and control of DR. This study attempted to comprehensively evaluate the relationship between dietary nutrient intake and the risk of DR to provide assistance for doctors in guiding the diet of diabetic patients. Data from eligible participants with diabetes from the US National Health and Nutrition Examination Survey (NHANES) from 2003–2018 were analyzed. Univariate logistic regression was used to assess the association between 58 dietary nutrient intakes and self-reported eye disease risk. Multivariate logistic regression model was used to further evaluate the relationship between the two groups after adjusting relevant confounding factors. A total of 4595 diabetic patients were included. People with self-reported eye affliction/retinopathy had lower dietary fiber, butanoic, octanoic, vitamin A, alpha-carotene, folate, magnesium, copper and caffeine intake compared to those without self-reported eye affliction/retinopathy. The pooled ORs (95% CIs) were 0.78 (0.62–0.98), 0.79 (0.63–0.99), 0.72 (0.58–0.91), 0.74 (0.59–0.93), 0.70 (0.55–0.88), 075 (0.60–0.95), 0.79 (0.64–0.99), 0.67 (0.54–0.84) and 0.80 (0.64–0.99). Dietary cholesterol and hexadecenoic intake were higher, with the pooled ORs (95% CIs) of 1.26 (1.01–1.58) and 1.27 (1.02–1.59), respectively. Our research found that among dietary nutrients, dietary fiber, butanoic, octanoic, vitamin A, alpha-carotene, folate, magnesium, copper and caffeine intake reduced the occurrence of DR. Cholesterol and hexadecenoic intake promoted the occurrence of DR. This suggests that certain dietary nutrients should be paid more attention in the prevention of DR.

## 1. Introduction

Diabetic ocular diseases are caused by chronic hyperglycemia in diabetic patients and include several ocular complications, including diabetic retinopathy (DR) and diabetic macular edema [1]. DR is the most common microvascular complication of diabetes and is one of the major causes of vision loss worldwide [2,3]. Without timely detection and early treatment, DR will progress from an early mild, non-proliferative disease to a severe proliferative disease [4]. Worldwide, the total prevalence of diabetes is 11.2%, and the estimated prevalence of DR in all diabetic adults aged 40 years and older is 34.6% (93 million people), of which the estimated prevalence of vision-threatening DR is 10.2% (28 million people). With increasing global trends in diabetes, the number of DR and vision-threatening retinopathy is expected to increase from 127 million and 37 million in 2010 to 191 million and 56 million in 2030 [5,6]. In the face of such situation, it is necessary to intervene in diabetic patients from multiple perspectives according to the epidemiological characteristics, such as regular eye examination, lifestyle adjustment, psychological intervention and daily diet management [7,8].

It is generally believed that the course of diabetes blood pressure and blood glucose level are the main risk factors for the progression of DR [8]. In addition, smoking, alcohol drinking, obesity, diet, high uric acid, high plasma fibrinogen and high homocysteine are also potential risk factors for DR [9,10]. A good lifestyle is critical for DR risk factor management; therefore, following a healthy lifestyle of reasonable diet, weight control and moderate exercise helps DR patients achieve a better prognosis [11]. Studies suggest that adherence to a Mediterranean diet and high intake of fruit, vegetables and fish may protect against the development of DR [12,13]. The diet contains a wide variety of nutrients, including macronutrients and micronutrients. It was found that dietary intake of carbohydrates, protein, dietary fiber, mono/polyunsaturated fatty acids, vitamins C, D, E, carotenoids, lutein, sodium, magnesium and other nutrients was closely related to the progression of DR [12,14,15]. However, the correlation between dietary nutrients and DR is inconsistent or even controversial in different studies. For example, in studies exploring the relationship between vitamin C intake and DR, a cohort study in Japan found that high vitamin C intake was associated with a 40% reduction in the risk of DR [16], while two other cross-sectional studies did not show any association between vitamin C intake and DR [14,17]. Therefore, our study used the population from the National Health and Nutrition Examination Survey (NHANES), a public database, to verify the correlation between dietary nutrients and eye affliction/retinopathy in individuals with diabetes, which may offer guidance on the diet control of the occurrence of DR.

## 2. Materials and Methods

### 2.1. Study Population

**Figure 1:**
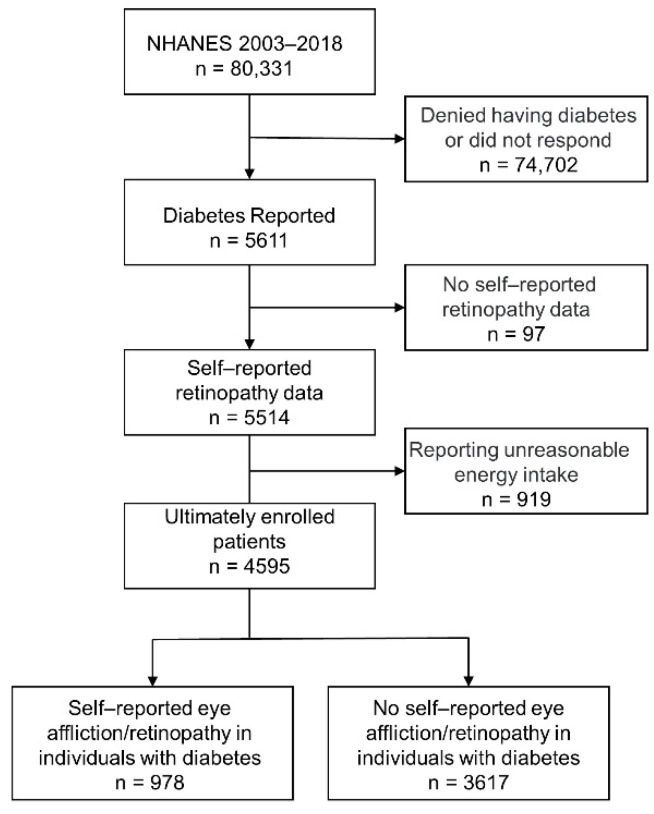
Study participant selection flowchart.

Data were gathered from the National Health and Nutrition Examination Survey (NHANES) 2003–2018. NHANES is an annual survey conducted by the National Center for Health Statistics of the Centers for Disease Control and Prevention (CDC) and is a cross-sectional survey of health and nutrition of United States civilians. Detailed description and data for each cycle of the NHANES is available on the official website (https://www.cdc.gov/nchs/nanes/index.htm (accessed on 6 October 2021)). The National Center approved study procedures for Health Statistics Research Ethics Review Board, informed consent were provided to participants before any data were collected, and details of the protocols for NHANES are available on the CDC website [18]. The selection of participants for the present study is schematically shown in Figure 1. In this dataset of 80,331 responders, 5611 individuals reported diabetes. We defined “diabetes” (*n* = 5611) as a response of “yes” to the following questions: “Other than during pregnancy, have you ever been told by a doctor or health professional that you have diabetes or sugar diabetes”? These cases were recruited in the NHANES over the 16-year period spanning 2003–2018 and had data on self-reported eye affliction/retinopathy in 4595 individuals.

### 2.2. Outcome and Covariates

Self-reported eye affliction/retinopathy was defined as an affirmative answer to the following question: “Has a doctor ever told you that diabetes has affected your eyes or that you had retinopathy”? The validity of the self-reported results of the study participants is questionable. Therefore, we evaluated the validity of this outcome indicator by using fundus photographs of patients reported in the NHANES 2005-208 dataset. Based on previous studies, covariates included age, sex, body mass index (BMI, weight divided by height squared) race and ethnicity, education level, smoking status, alcohol use status, hypertension and duration of diabetes [19].

### 2.3. Ascertainment of Diet

A total of 58 dietary nutrients were included in the study, and the intakes of them were obtained from the total nutrient intake file, which contains total nutrients for all foods and beverages. All participants were eligible for two 24 h dietary recalls. The first dietary recall interview was collected in-person in the mobile examination center, and the second interview was collected by telephone 3–10 days later. Our analysis will use the average consumption of both recalls.

### 2.4. Statistical Analysis

Participants were grouped according to outcome indicators. Differences in baseline characteristics between groups were compared by t-tests for continuous variables and χ2 tests for categoric variables. If the continuous variable did not follow a normal distribution, the data was logarithmically transformed. Data were expressed as mean ± standard deviation of continuous variables and numbers (percentages) of categorical variables. If the continuous variable did not follow a normal distribution, data were expressed as Median (interquartile range). In logistic analysis, features with three or more categories are treated as indicative (dummy) variables. Using logistic regression to calculate the OR and 95% CI for the prevalence of eye affliction/retinopathy in individuals with diabetes per quintile of laboratory test indicators or dietary nutrient intake, we calculated four different logistic regression models. Model 1 was a rough model, and Model 2 was adjusted for age (continuous) and sex. Model 3 included covariates of Model 2, with additional adjustments for BMI (continuous), race (Mexican American, non-Hispanic black, non-Hispanic white and other RACES), degree of education (less than high school, high school/high school graduate, college/university graduate, college or above) and diabetes (continuous). Model 4 included covariates of Model 3 with additional adjustments for hypertension, smoking (current, quit, never smoking) and alcohol consumption. Given the complex, multistage probability cluster design of NHANES, weighting took into account several features of the survey: the differential probabilities of selection for the individual domains, non-response to survey instruments and differences between the final sample and the U.S. civilian non-institutionalized population. In this analysis, we combined eight 2-year cycles to produce estimates with greater precision and smaller sampling error. New multi-year sample weight was computed by simply dividing the 2-year sample weights by the number (eight in this analysis) of 2-year cycles in this analysis [18]. R 4.0.3 (R Foundation for Statistical Computing, Vienna, Austria) was used, and *p* ≤ 0.05 was considered statistically significant.

## 3. Results

### 3.1. Characteristics of Participants

Baseline data and characteristics of participants are summarized in Table 1. According to a rigorous participant screening process, 4595 participants were eventually included, including 2288 men (49.79%) and 2307 women (50.21%). The average age of the total samples was 62.19 ± 13.20 years. A total of 978 participants self-reported “eye affliction/retinopathy in individuals with diabetes”. Compared with diabetic patients without self-reported eye affliction/retinopathy, there were no significant differences in age, sex, blood pressure (systolic and diastolic blood pressure), BMI, smoking and drinking between patients with self-reported eye affliction/retinopathy, but there were significant differences in duration of diabetes, insulin use, race and education level between the two groups.

### 3.2. Macrodietary Nutrients

Univariate logistic regression analysis was shown in Table 2 to assess dietary macronutrients in diabetic patients with self-reported eye affliction/retinopathy. The results showed that energy, protein, moisture, carbohydrate and total sugars intake were not correlated with eye affliction/retinopathy. Dietary fiber intake reduces the risk of eye affliction/retinopathy, and the pooled OR (95% CI) was 0.78 (0.62–0.98), comparing the highest with the lowest quintiles. After multivariable adjustment for potential confounders, such as age, sex, duration of diabetes, etc., the association weakened but remained significant, the pooled ORs (95% CIs) across increasing quintiles of dietary fiber were 1.35 (0.99–1.84), 0.92 (0.66–1.29), 0.82 (0.58–1.15), and 0.75 (0.52–1.07; *p* trend = 0.006) (Table 3).

### 3.3. Dietary Fatty Acids

Although dietary intake of total fat, total saturated fatty acids, total monounsaturated fatty acids and total polyunsaturated fatty acids was not associated with risk of eye affliction/retinopathy, some individual dietary fatty acids were associated with the risk of eye affliction/retinopathy (Table 4). Butanoic and octanoic of saturated fatty acid (SFA) intake reduces the risk of eye affliction/retinopathy; the pooled ORs (95% CIs) were 0.79 (0.63–0.99) and 0.72 (0.58–0.91), comparing the second with the lowest quintiles. Hexadecenoic of monounsaturated fatty acids (MFA) intake increases the risk of eye affliction/retinopathy; the pooled OR (95% CI) were 1.27 (1.02–1.59), comparing the highest with the lowest quintiles. These associations weakened after adjustment for confounders, such as age, sex, and duration of diabetes, etc. (Table 3).

### 3.4. Dietary Vitamins

Univariate logistic regression analysis assessed the association between dietary vitamins and self-reported eye affliction/retinopathy in diabetic patients (Table 5). Most dietary vitamin intake is not associated with the risk of eye distress /retinopathy. However, vitamin A, alpha-carotene (α-carotene) and dietary folate equivalent seem to be associated with the risk of eye distress /retinopathy. Further analysis of the association showed that vitamin A, α-carotene and dietary folate equivalent intake reduces the risk of eye affliction/retinopathy. However, after adjusting for confounding factors, such as age, sex, duration of diabetes, etc., the association remained significant for vitamin A (Table 3).

### 3.5. Formatting of Mathematical Components

The dietary micronutrients evaluated contain a wide range of metals (sodium, potassium, calcium, magnesium, iron, copper and zinc), phosphorus, selenium and caffeine (a component of coffee and tea). Univariate logistic regression analysis showed that dietary magnesium intake reduces the risk of eye affliction/retinopathy, and the pooled OR (95% CI) was 0.79 (0.64–0.99), comparing the highest with the lowest quintiles. Dietary copper intake is similar to that of magnesium (OR = 0.67, 95% CI: 0.54–0.84) (Table 6). After multivariable adjustment for potential confounding factors, such as age, sex, duration of diabetes, etc., the inverse association was attenuated (Table 3). Similarly, dietary caffeine intake reduces the risk of eye affliction/retinopathy with diabetes.

## 4. Discussion

To our knowledge, this is the first cross-sectional study to examine the association between dietary intake of nearly all nutrients and the risk of DR. Our study adopted the source from NHANES, a public database, to verify the correlation between dietary nutrients and eye affliction/retinopathy in individuals with diabetes. We eventually enrolled 4,595 diabetics and divided the participants into two groups based on whether they had self-reported eye affliction/retinopathy, through a rigorous screening process. At the same time, we screened a total of 58 dietary nutrients of four types, including macro-dietary nutrients (7), dietary fatty acids (23), dietary vitamins (18) and dietary trace elements (10). Univariate and multivariate logistic regression were used to assess the association between each dietary nutrient intake and the risk of eye affliction/retinopathy. The results showed that dietary fiber, butanoic, octanoic, vitamin a, α-carotene, folate, magnesium, copper and caffeine intake were negatively correlated with the risk of eye affliction/retinopathy. Cholesterol and hexadecenoic intake were positively correlated with the risk of eye affliction/retinopathy. Multivariate logistic analysis showed that after adjusting for age, gender and duration of diabetes, dietary fiber, cholesterol, octanoic, hexadecenoic, vitamin A, magnesium and copper intake still were correlated with the risk of eye affliction/retinopathy.

Studies on the relationship between dietary fiber intake and DR are still controversial. One Spanish study showed no association between dietary fiber intake and DR [20]. Results of a cross-sectional study in India showed that subjects consuming a low-fiber diet had a higher risk of DR than those consuming a healthy fiber diet (OR = 1.21, 95% CI: 1.02–1.94) [21]. Of course, different study results may be related to different dietary habits in different regions, and we based our findings on the premise that dietary fiber intake can affect the occurrence of DR on NHANSE. Studies have shown that dietary fiber promotes beneficial physiological effects such as defecation, lowered blood cholesterol and post-prandial glucose regulation [22,23,24,25]. High-fiber diets not only improve diabetes control, but also reduce insulin requirements and the incidence of complications [26,27,28].

Our study showed that dietary intake of total fat, total SFA, total MFA and total PFA was not associated with DR, but the increased intake of single fatty acids reduced the risk of DR, which was consistent with the findings of Alcubierre et al. [20]. However, Hayes et al. [29] observed that single fatty acid intake increased the risk of DR. Many previous studies have examined the relationship between PFA and DR, and the results showed that the intake of PFA could help prevent retinopathy [30,31]. PFA is one of the ligands activated by the peroxisome proliferator-activated receptor α (PPAR-α) [32], a nuclear receptor protein that inhibits the vascular endothelial growth factor (VEGF) pathway [33]. It has been presumed that PFA improves dyslipidemia and increases the activity of PPAR-α, which may partly explain why some studies have linked increased PFA intake with a reduced likelihood of developing DR [34].

Our study found that dietary vitamin intake (such as vitamin A, α -carotene and folic acid) in patients with DR was lower than that in non-DR patients with diabetes, and previous studies rarely focused on the dietary vitamin A intake status in patients with DR. Antioxidants, vitamin A and α-carotene are thought to prevent oxidant-mediated inflammation by scavenging reactive oxygen species (ROS) and inhibiting the activation of nuclear factor kappa B(NF-κB). Nuclear factor kappa B is a transcription factor that promotes the expression of genes that induce inflammation [35,36,37]. Oxidative stress and inflammation are closely related to the occurrence and progression of DR [38,39]. Folate has been widely used in clinical treatment of DR. Many clinical studies have reported extremely low plasma levels of folate in patients with proliferative or nonproliferative diabetic retinopathy [40]. Correspondingly, folate has a protective effect on DR by inhibiting angiogenesis, inflammation and oxidative stress [38,41]. Studies on the relationship between DR and the intake of other vitamin nutrients, such as vitamins C, D, E and other carotenoids, have been inconsistent. One systematic review indicated that vitamin C and D had a positive or no effect for risk of DR, and vitamin E had a negative or no effect [12].

Previous studies confirm our conclusion that magnesium and copper are essential dietary micronutrients, and their deficiency can lead to a range of dysfunctions related to glucose metabolism. Studies have shown that hypomagnesemia is a risk factor for diabetic retinopathy [42]. Magnesium deficiency can lead to pro-inflammatory and pro-fibrotic reactions and oxidative stress due to the reduction of certain protective enzymes containing magnesium [43,44,45]. Magnesium also acts as a cofactor of the glucose transport mechanism in cell membrane, helping carbohydrate oxidase and insulin secretion, binding and activity [46]. Oral magnesium supplementation improves insulin sensitivity and metabolic control in diabetic patients with low serum magnesium levels [47]. Similarly, copper deficiency leads to decreased activity of oxidative defense enzyme, copper zinc superoxide dismutase and selenium-dependent glutathione peroxidase, and reactive oxygen scavengers such as glutathione and metallothionein alter the oxidative defense system, leading to excessive oxidative stress and tissue damage [48]. Plausible explanations for why caffeine may reduce the risk of DR are that, on the one hand, caffeine protects against external BRB damage by inhibiting apoptotic cell death induced by hyperglycemic/hypoxic injury [49]. On the other hand, by inhibiting the expression of ROS-induced VEGF, caffeine shows antioxidant and potential anti-angiogenic activities on retinal endothelial cells and retinal neovascular, respectively [50,51]; thus, it may delay the occurrence and progression of DR.

The advantages of this study include large sample size, comprehensive dietary nutrients, and NHANES is well established and designed to be representative of the US population, so our results are broadly applicable. Our study also has several limitations: First, the study design and data are cross sectional, so we can only obtain the correlation between the two but cannot infer the accurate causal relationship. Second, the dietary data recalled twice in 24 h may not accurately represent the normal diet for a long time, during which the disease may develop, and participants may change their eating habits after collecting dietary information. Third, the outcome measures were self-reported eye disease/retinopathy in diabetic patients, which may deviate from the DR discussed. However, we used NHANES 2005-2008 datasets with ocular fundus photographic to verify that self-reported outcome indicators and DR had certain validity.

## 5. Conclusions

In conclusion, compared with those who did not self-report eye affliction/retinopathy with diabetes, dietary nutrients, cholesterol and hexadecenoic intake were associated with a higher risk of eye affliction/retinopathy, and dietary fiber, butanoic, octanoic, vitamin a, alpha-carotene, folate, magnesium, copper and caffeine intake were associated with a lower risk of eye affliction/retinopathy. Of course, these findings need to be confirmed by prospective studies, and further studies are needed to explore the correlation between dietary nutrient intake and the risk of DR progression.

## Figures and Tables

**Table 1:** Baseline data of study subjects.

	Total *n* = 4595	Self-Reported Eye Affliction/Retinopathy in Individuals with Diabetes		t/χ^2^	*p*-Value ^a^
		Yes *n* = 978	No *n* = 3617		
Male, *n* (%)	2288 (49.79)	497 (50.82)	1791 (49.52)	0.52	0.470
Age (year, $\bar{x}$ ± s)	62.19 ± 13.20	62.62 ± 12.18	62.08 ± 13.45	1.15	0.252
Duration of diabetes (year, $\bar{x}$ ± s)	12.23 ± 11.76	16.90 ± 12.80	10.96 ± 11.14	14.28	<0.0001
BMI (kg/m^2^, $\bar{x}$ ± s)	32.30 ± 7.52	32.61 ± 7.84	32.22 ± 7.43	1.42	0.157
SBP (mmHg, $\bar{x}$ ± s)	132.22 ± 20.57	133.05 ± 21.76	131.99 ± 20.22	1.40	0.161
DBP (mmHg, $\bar{x}$ ± s)	68.34 ± 13.27	67.70 ± 13.14	68.51 ± 13.30	−1.67	0.095
Hypertension, *n* (%)	3196 (69.55)	697 (71.27)	2499 (69.09)	1.72	0.189
Taking insulin now, *n* (%)	1294 (28.16)	489 (50.00)	805 (22.26)	284.90	<0.0001
Smoke, *n* (%)				0.52	0.770
Smoking now	597 (12.99)	124 (12.68)	473 (13.08)		
Cigarettes	1674 (36.43)	368 (37.63)	1306 (36.11)		
Never	2295 (49.95)	485 (49.59)	1810 (50.04)		
Drinks, *n* (%)	1304 (28.39)	288 (29.45)	1016 (28.09)	0.70	0.403
Race, *n* (%)				23.48	<0.0001
Non-Hispanic whites	1636 (35.60)	319 (32.62)	1317 (36.41)		
Non-Hispanic black	1251 (27.23)	266 (27.20)	985 (27.23)		
Mexican American	876 (19.06)	189 (19.33)	687 (18.99)		
Other race	832 (18.11)	204 (20.86)	628 (17.36)		
Education Level, *n* (%)				12.72	0.005
Less than high school	868 (18.89)	213 (21.78)	655 (18.11)		
High school	1810 (39.39)	394 (40.29)	1416 (39.15)		
College	1203 (26.18)	248 (25.36)	955 (26.40)		
College above	705 (15.34)	122 (12.47)	583 (16.12)		
C-reactive protein (mg/dL) ^b^	0.29 (0.12–0.65)	0.28 (0.13–0.62)	0.29 (0.12–0.65)	0.91	0.365
HDL—Cholesterol (mmol/L) ^b^	1.19 (1.01–1.45)	1.19 (1.01–1.45)	1.19 (1.01–1.45)	0.86	0.392
LDL—Cholesterol (mmol/L) ^b^	2.48 (1.91–3.18)	2.37 (1.89–3.18)	2.51 (1.91–3.18)	−0.59	0.553
Total Cholesterol (mmol/L) ^b^	4.58 (3.88–5.40)	4.55 (3.90–5.40)	4.58 (3.88–5.40)	0.08	0.939
Triglyceride (mmol/L) ^b^	1.43 (1.01–2.10)	1.42 (1.01–2.13)	1.43 (1.02–2.09)	−0.41	0.685
Glycohemoglobin (%) ^b^	6.90 (6.20–8.10)	7.20 (6.30–8.40)	6.90 (6.20–8.00)	3.59	<0.0001
Albumin, urine (mg/L) ^b^	15.05 (6.43–50.00)	20.50 (7.83–77.13)	14.00 (6.20–42.70)	6.21	<0.0001
Creatinine, urine (mg/dL) ^b^	100.00(62.00–149.00)	96.00(61.00–142.00)	101.00(63.00–151.00)	−1.40	0.161

BMI, body mass index; SBP, systolic blood pressure; DBP, diastolic blood pressure; $\bar{x}$ ± s, mean ± standard deviation; IQR, interquartile range. ^a^ This is a comparison of self-reported eye affliction/retinopathy in individuals with diabetes; ^b^ Data do not follow a normal distribution, are expressed as median (IQR) and will be logarithmically transformed.

**Table 2:** Univariate logistic regression analysis of the association of macrodietary nutrients with self-reported eye affliction/retinopathy in individuals with diabetes.

	Quintile of Macrodietary Nutrients, OR (95% CI)				
	1	2	3	4	5
Energy (kcal)	1.00	1.04 (0.84–1.30)	1.00 (0.80–1.25)	0.82 (0.65–1.03)	0.98 (0.79–1.23)
Protein (gm)	1.00	1.16 (0.93–1.45)	1.03 (0.82–1.29)	1.00 (0.79–1.26)	1.18 (0.95–1.48)
Carbohydrate (gm)	1.00	0.98 (0.79–1.21)	0.92 (0.74–1.14)	0.81 (0.65–1.01)	0.82 (0.66–1.03)
Total sugars (gm)	1.00	1.11 (0.90–1.38)	0.81 (0.65–1.02)	0.93 (0.74–1.16)	0.81 (0.64–1.01)
Dietary fiber (gm)	1.00	1.11 (0.90–1.38)	0.97 (0.78–1.21)	0.80 (0.64–1.00)	0.78 (0.62–0.98)
Cholesterol (mg)	1.00	1.19 (0.95–1.49)	1.03 (0.82–1.30)	0.98 (0.77–1.23)	1.26 (1.01–1.58)
Moisture (gm)	1.00	1.12 (0.90–1.39)	0.90 (0.72–1.13)	0.96 (0.77–1.21)	1.01 (0.80–1.26)

**Table 3:** Multiple logistic regression analysis of association of dietary nutrients with self-reported eye affliction/retinopathy in individuals with diabetes.

	Quintile of Dietary Nutrients, OR (95% CI)					*p* Trend
	1	2	3	4	5	
Dietary fiber (gm)						
Model 1	1.00	1.11 (0.90–1.37)	0.96 (0.77–1.20)	0.80 (0.63–0.99)	0.77 (0.61–0.96)	0.001
Model 2	1.00	1.35 (0.99–1.84)	0.92 (0.66–1.29)	0.82 (0.58–1.15)	0.75 (0.52–1.07)	0.006
Model 3	1.00	1.35 (0.97–1.85)	0.99 (0.74–1.30)	0.85 (0.61–1.17)	0.74 (0.49–1.01)	0.016
Model 4	1.00	1.34 (0.96–1.85)	1.03 (0.73–1.46)	0.85 (0.59–1.21)	0.80 (0.55–1.16)	0.034
Cholesterol (mg)						
Model 1	1.00	1.18 (0.95–1.48)	1.03 (0.82–1.30)	0.97 (0.77–1.23)	1.27 (1.01–1.59)	0.273
Model 2	1.00	1.44 (1.04–1.98)	0.98 (0.70–1.39)	1.10 (0.78–1.55)	1.64 (1.18–2.29)	0.054
Model 3	1.00	1.19 (0.94–1.49)	1.00 (0.80–1.26)	1.06 (0.84–1.33)	1.28 (1.01–1.61)	0.153
Model 4	1.00	1.46 (1.04–2.05)	1.04 (0.72–1.49)	1.13 (0.79–1.62)	1.52 (1.07–2.16)	0.138
SFA 4:0 (Butanoic) (gm)						
Model 1	1.00	0.79 (0.63–0.99)	0.93 (0.67–1.04)	1.05 (0.85–1.30)	0.86 (0.68–1.07)	0.903
Model 2	1.00	0.72 (0.52–1.00)	0.83 (0.60–1.15)	1.14 (0.83–1.57)	0.82 (0.58–1.16)	0.885
Model 3	1.00	0.89 (0.71–1.13)	0.94 (0.74–1.18)	1.17 (0.93–1.48)	0.92 (0.73–1.17)	0.160
Model 4	1.00	0.77 (0.54–1.09)	0.85 (0.61–1.20)	1.23 (0.88–1.72)	0.91 (0.63–1.31)	0.515
SFA 8:0 (Octanoic) (gm)						
Model 1	1.00	0.73 (0.58–0.91)	0.89 (0.71–1.10)	0.94 (0.75–1.16)	0.92 (0.74–1.14)	0.841
Model 2	1.00	0.74 (0.53–1.03)	0.86 (0.62–1.18)	0.92 (0.66–1.28)	1.02 (0.73–1.42)	0.599
Model 3	1.00	0.75 (0.59–0.94)	0.89 (0.71–1.13)	0.93 (0.74–1.18)	0.94 (0.74–1.19)	0.117
Model 4	1.00	0.74 (0.52–1.05)	0.84 (0.60–1.19)	0.93 (0.66–1.33)	1.05 (0.74–1.49)	0.516
MFA 16:1 (Hexadecenoic) (gm)						
Model 1	1.00	1.11 (0.88–1.39)	1.10 (0.88–1.38)	0.96 (0.76–1.22)	1.27 (1.02–1.60)	0.160
Model 2	1.00	1.14 (0.83–1.56)	1.07 (0.76–1.50)	1.14 (0.82–1.60)	1.21 (0.84–1.73)	0.349
Model 3	1.00	1.10 (0.87–1.38)	1.12 (0.90–1.42)	0.96 (0.76–1.21)	1.40 (1.10–1.78)	0.018
Model 4	1.00	1.20 (0.86–1.67)	1.03 (0.72–1.46)	1.17 (0.82–1.66)	1.16 (0.79–1.69)	0.525
Vitamin A (mcg)						
Model 1	1.00	0.93 (0.75–1.15)	0.82 (0.65–1.02)	0.92 (0.74–1.14)	0.73 (0.58–0.92)	0.016
Model 2	1.00	0.86 (0.62–1.19)	1.02 (0.73–1.41)	1.00 (0.73–1.37)	0.68 (0.48–0.96)	0.157
Model 3	1.00	1.01 (0.80–1.28)	0.81 (0.65–1.00)	1.02 (0.81–1.28)	0.78 (0.62–0.98)	0.043
Model 4	1.00	0.84 (0.60–1.19)	1.03 (0.73–1.45)	1.02 (0.73–1.42)	0.70 (0.48–1.02)	0.278
Alpha-carotene, (mcg)						
Model 1	1.00	1.03 (0.83–1.27)	0.69 (0.55–0.87)	0.92 (0.74–1.14)	0.92 (0.71–1.14)	0.234
Model 2	1.00	1.35 (0.97–1.88)	0.96 (0.68–1.36)	1.16 (0.83–1.64)	1.05 (0.75–1.48)	0.861
Model 3	1.00	1.12 (0.80–1.41)	0.73 (0.58–0.92)	0.99 (0.79–1.26)	0.95 (0.75–1.19)	0.224
Model 4	1.00	1.35 (0.95–1.91)	0.87 (0.60–1.26)	1.13 (0.79–1.62)	1.12 (0.78–1.59)	0.948
Folate, DFE (mcg)						
Model 1	1.00	0.84 (0.67–1.04)	0.96 (0.77–1.19)	0.75 (0.59–0.94)	0.83 (0.66–1.04)	0.055
Model 2	1.00	0.80 (0.58–1.11)	0.87 (0.63–1.19)	0.77 (0.55–1.07)	0.96 (0.69–1.33)	0.611
Model 3	1.00	0.89 (0.71–1.13)	1.10 (0.87–1.39)	0.80 (0.64–1.01)	0.89 (0.70–1.21)	0.066
Model 4	1.00	0.80 (0.57–1.12)	0.88 (0.63–1.22)	0.82 (0.58–1.17)	1.00 (0.71–1.42)	0.921
Magnesium (mg)						
Model 1	1.00	0.94 (0.76–1.17)	0.90 (0.72–1.12)	0.76 (0.60–0.95)	0.78 (0.62–0.98)	0.006
Model 2	1.00	1.06 (0.77–1.44)	0.88 (0.64–1.21)	0.76 (0.54–1.07)	0.78 (0.54–1.11)	0.040
Model 3	1.00	0.98 (0.78–1.24)	0.98 (0.77–1.23)	0.77 (0.56–1.08)	0.78 (0.54–1.01)	0.032
Model 4	1.00	1.12 (0.81–1.55)	0.96 (0.69–1.35)	0.86 (0.60–1.22)	0.77 (0.53–1.12)	0.079
Copper (mg)						
Model 1	1.00	0.73 (0.59–0.91)	0.83 (0.67–1.02)	0.72 (0.58–0.89)	0.66 (0.52–0.82)	0.001
Model 2	1.00	0.72 (0.53–0.98)	0.77 (0.57–1.05)	0.59 (0.42–0.83)	0.64 (0.45–0.91)	0.003
Model 3	1.00	0.74 (0.56–0.97)	0.86 (0.68–1.08)	0.61 (0.44–0.90)	0.68 (0.44–0.89)	0.011
Model 4	1.00	0.77 (0.56–1.07)	0.83 (0.60–1.15)	0.63 (0.45–0.90)	0.70 (0.49–1.01)	0.019
Caffeine (mg)						
Model 1	1.00	0.80 (0.64–0.99)	0.95 (0.76–1.18)	0.86 (0.69–1.07)	0.88 (0.71–1.10)	0.441
Model 2	1.00	0.65 (0.47–0.90)	0.99 (0.72–1.34)	0.87 (0.62–1.21)	0.87 (0.61–1.24)	0.837
Model 3	1.00	0.83 (0.66–1.04)	0.99 (0.79–1.25)	0.84 (0.67–1.05)	0.87 (0.69–1.10)	0.331
Model 4	1.00	0.67 (0.47–0.95)	1.01 (0.73–1.41)	0.80 (0.56–1.13)	0.96 (0.66–1.39)	0.977

SFA, saturated fatty acids; MFA, monounsaturated fatty acids; DFE, dietary folate equivalent; Model 1: adjusted for sex and age; Model 2: adjusted for sex, age, BMI, race, educational level, smoking, and alcohol; Model 3: adjusted for sex, age, BMI, race, educational level, smoking, alcohol, the duration of diabetes; Model 4: adjusted for sex, age, BMI, race, educational level, smoking, alcohol, the duration of diabetes, hypertension, glycohemoglobin (HbA1c).

**Table 4:** Univariate logistic regression analysis of association of dietary fatty acids with self-reported eye affliction/retinopathy in individuals with diabetes.

	Quintile of Dietary Fatty Acids, OR (95% CI)				
	1	2	3	4	5
Total fat (gm)	1.00	0.93 (0.74–1.16)	1.05 (0.84–1.31)	1.01 (0.81–1.26)	0.99 (0.79–1.23)
Total saturated fatty acids (gm)	1.00	0.98 (0.79–1.23)	1.04 (0.83–1.30)	0.95 (0.76–1.19)	1.06 (0.85–1.33)
SFA 4:0 (Butanoic) (gm)	1.00	0.79 (0.63–0.99)	0.83 (0.67–1.04)	1.05 (0.85–1.30)	0.86 (0.69–1.07)
SFA 6:0 (Hexanoic) (gm)	1.00	0.84 (0.67–1.05)	0.98 (0.79–1.23)	1.09 (0.88–1.36)	0.96 (0.77–1.20)
SFA 8:0 (Octanoic) (gm)	1.00	0.72 (0.58–0.91)	0.88 (0.71–1.10)	0.94 (0.75–1.16)	0.92 (0.74–1.14)
SFA 10:0 (Decanoic) (gm)	1.00	0.82 (0.65–1.03)	0.98 (0.79–1.22)	1.02 (0.82–1.26)	0.97 (0.78–1.21)
SFA 12:0 (Dodecanoic) (gm)	1.00	0.85 (0.68–1.06)	0.98 (0.79–1.22)	0.80 (0.64–1.01)	1.02 (0.82–1.27)
SFA 14:0 (Tetradecanoic) (gm)	1.00	0.88 (0.70–1.01)	0.94 (0.75–1.18)	1.13 (0.91–1.40)	1.02 (0.81–1.27)
SFA 16:0 (Hexadecanoic) (gm)	1.00	0.97 (0.77–1.21)	1.04 (0.83–1.30)	0.95 (0.76–1.19)	1.11 (0.89–1.38)
SFA 18:0 (Octadecanoic) (gm)	1.00	0.97 (0.77–1.21)	1.00 (0.80–1.25)	0.94 (0.75–1.18)	1.03 (0.83–1.29)
Total monounsaturated fatty acids (gm)	1.00	0.91 (0.72–1.14)	1.08 (0.87–1.35)	1.00 (0.80–1.25)	0.97 (0.78–1.21)
MFA 16:1 (Hexadecenoic) (gm)	1.00	1.11 (0.89–1.39)	1.11 (0.88–1.39)	0.96 (0.76–1.22)	1.27 (1.02–1.59)
MFA 18:1 (Octadecenoic) (gm)	1.00	0.99 (0.79–1.24)	1.10 (0.88–1.37)	1.04 (0.83–1.30)	0.95 (0.76–1.19)
MFA 20:1 (Eicosenoic) (gm)	1.00	0.91 (0.73–1.14)	0.85 (0.68–1.07)	1.19 (0.96–1.47)	0.86 (0.68–1.07)
MFA 22:1 (Docosenoic) (gm)	1.00	1.14 (0.91–1.42)	1.14 (0.91–1.43)	1.13 (0.90–1.41)	1.13 (0.91–1.42)
Total polyunsaturated fatty acids (gm)	1.00	1.01 (0.81–1.26)	1.06 (0.85–1.32)	1.18 (0.95–1.47)	0.90 (0.71–1.13)
PFA 18:2 (Octadecadienoic) (gm)	1.00	0.98 (0.78–1.23)	1.03 (0.83–1.29)	1.20 (0.97–1.49)	0.86 (0.68–1.08)
PFA 18:3 (Octadecatrienoic) (gm)	1.00	0.99 (0.79–1.24)	1.19 (0.96–0.48)	1.08 (0.87–1.35)	0.92 (0.73–1.15)
PFA 18:4 (Octadecatetraenoic) (gm)	1.00	1.01 (0.78–1.31)	0.98 (0.80–1.20)	1.07 (0.87–1.32)	0.98 (0.80–1.19)
PFA 20:4 (Eicosatetraenoic) (gm)	1.00	0.99 (0.79–1.24)	0.95 (0.76–1.19)	0.92 (0.73–1.15)	1.10 (0.88–1.36)
PFA 20:5 (Eicosapentaenoic) (gm)	1.00	1.02 (0.81–1.28)	1.19 (0.95–1.48)	1.19 (0.95–1.49)	1.10 (0.88–1.38)
PFA 22:5 (Docosapentaenoic) (gm)	1.00	0.92 (0.74–1.15)	1.10 (0.89–1.37)	0.89 (0.71–1.11)	0.95 (0.76–1.18)
PFA 22:6 (Docosahexaenoic) (gm)	1.00	1.11 (0.89–1.39)	0.88 (0.69–1.11)	1.16 (0.93–1.45)	1.03 (0.82–1.29)

SFA, saturated fatty acids; MFA, monounsaturated fatty acids; PFA, polyunsaturated fatty acids.

**Table 5:** Univariate logistic regression analysis of association of dietary vitamins with self-reported eye affliction/retinopathy in individuals with diabetes.

	Quintile of Dietary Vitamins, OR (95% CI)				
	1	2	3	4	5
Vitamin E as alpha-tocopherol (mg)	1.00	0.98 (0.79–1.22)	1.06 (0.85–1.32)	0.93 (0.75–1.16)	0.81 (0.64–1.02)
Retinol (mcg)	1.00	1.23 (0.99–1.52)	0.94 (0.75–1.18)	0.94 (0.75–1.18)	1.03 (0.83–1.29)
Vitamin A (mcg)	1.00	0.93 (0.75–1.15)	0.83 (0.66–1.03)	0.93 (0.75–1.15)	0.74 (0.59–0.93)
Alpha-carotene (mcg)	1.00	1.03 (0.83–1.28)	0.70 (0.55–0.88)	0.93 (0.75–1.16)	0.93 (0.74–1.15)
Beta-carotene (mcg)	1.00	1.01 (0.81–1.26)	1.01 (0.81–1.25)	0.98 (0.80–1.23)	0.81 (0.65–1.02)
Beta-cryptoxanthin (mcg)	1.00	0.90 (0.72–1.13)	1.10 (0.88–1.37)	0.97 (0.77–1.21)	0.95 (0.76–1.19)
Lycopene (mcg)	1.00	0.78 (0.62–0.97)	0.73 (0.58–0.91)	0.89 (0.72–1.10)	0.85 (0.69–1.06)
Lutein + zeaxanthin (mcg)	1.00	1.03 (0.83–1.28)	0.96 (0.77–1.20)	1.01 (0.81–1.25)	0.83 (0.66–1.04)
Thiamin (Vitamin B1) (mg)	1.00	1.07 (0.86–1.34)	1.03 (0.82–1.28)	1.05 (0.84–1.31)	0.87 (0.69–1.10)
Riboflavin (Vitamin B2) (mg)	1.00	0.90 (0.72–1.26)	0.94 (0.75–1.17)	0.94 (0.76–1.18)	0.92 (0.74–1.15)
Niacin (mg)	1.00	0.94 (0.75–1.17)	0.97 (0.77–1.21)	1.02 (0.82–1.27)	0.94 (0.75–1.18)
Vitamin B6 (mg)	1.00	0.98 (0.79–1.22)	0.97 (0.78–0.21)	0.88 (0.70–1.10)	0.91 (0.73–1.15)
Folate, DFE (mcg)	1.00	0.85 (0.68–1.06)	0.97 (0.78–1.20)	075 (0.60–0.95)	0.84 (0.67–1.05)
Total choline (mg)	1.00	1.20 (0.97–1.50)	0.88 (0.70–1.11)	0.99 (0.78–1.24)	1.16 (0.93–1.45)
Vitamin B12 (mcg)	1.00	0.80 (0.94–0.99)	0.94 (0.76–1.17)	0.92 (0.74–1.14)	0.86 (0.69–1.07)
Vitamin C (mg)	1.00	1.08 (0.87–1.34)	1.03 (0.83–1.29)	1.01 (0.81–1.26)	0.85 (0.68–1.07)
Vitamin D (D2 + D3) (mcg)	1.00	1.10 (0.88–1.37)	0.99 (0.79–1.24)	1.03 (0.83–1.30)	1.00 (0.80–1.25)
Vitamin K (mcg)	1.00	1.06 (0.85–1.33)	1.10 (0.89–1.38)	0.98 (0.78–1.22)	0.84 (0.66–1.06)

DFE, dietary folate equivalent.

**Table 6:** Univariate logistic regression analysis of the association of dietary micronutrients with self-reported eye affliction/retinopathy in individuals with diabetes.

	Quintile of Dietary Micronutrients, OR (95% CI)				
	1	2	3	4	5
Calcium (mg)	1.00	0.94 (0.76–1.18)	0.97 (0.78–1.20)	0.92 (0.73–1.15)	0.93 (0.74–1.16)
Phosphorus (mg)	1.00	1.05 (0.84–1.31)	0.92 (0.74–1.16)	0.98 (0.79–1.23)	0.98 (0.79–1.23)
Magnesium (mg)	1.00	0.95 (0.77–1.17)	0.91 (0.73–1.13)	0.77 (0.61–0.96)	0.79 (0.64–0.99)
Iron (mg)	1.00	1.16 (0.93–1.44)	1.12 (0.90–1.40)	0.99 (0.79–1.24)	0.89 (0.70–1.12)
Zinc (mg)	1.00	0.95 (0.76–1.18)	0.84 (0.67–1.05)	0.97 (0.78–1.21)	0.94 (0.76–1.18)
Copper (mg)	1.00	0.74 (0.60–0.92)	0.84 (0.68–1.03)	0.73 (0.58–0.90)	0.67 (0.54–0.84)
Sodium (mg)	1.00	0.95 (0.76–1.18)	0.93 (0.74–1.16)	0.93 (0.74–1.16)	1.02 (0.82–1.26)
Potassium (mg)	1.00	1.01 (0.82–1.26)	0.88 (0.70–1.10)	0.87 (0.69–1.08)	0.90 (0.72–1.12)
Selenium (mcg)	1.00	1.01 (0.81–1.26)	1.04 (0.83–1.30)	0.98 (0.78–1.23)	1.08 (0.86–1.34)
Caffeine (mg)	1.00	0.80 (0.64–0.99)	0.95 (0.76–1.18)	0.87 (0.69–1.08)	0.89 (0.72–1.11)

## Data Availability

The data that support the findings of this study are available from the corresponding author upon reasonable request.
